# Surface Acoustic Wave DMMP Gas Sensor with a Porous Graphene/PVDF Molecularly Imprinted Sensing Membrane

**DOI:** 10.3390/mi12050552

**Published:** 2021-05-12

**Authors:** Sheng Xu, Rui Zhang, Junpeng Cui, Tao Liu, Xiuli Sui, Meng Han, Fu Zheng, Xiaoguang Hu

**Affiliations:** 1 School of Software and Communication, Tianjin Sino-German University of Applied Sciences, Tianjin 300350, China; xusheng1025@126.com (S.X.); hitchcockzhr@163.com (R.Z.); cuijunpengok@163.com (J.C.); liutao_80@163.com (T.L.); suixl08@163.com (X.S.); 2 School of Electrical and Information Engineering, Tianjin University, Tianjin 300072, China; 3 Deepinfar Ocean Technology Co., Ltd., Building #28, Tianjin Binhai Innovation Park, Tianjin 300000, China; cart2008@163.com; 4 Tianjin Key Laboratory of Film Electronic and Communicate Devices, School of Electrical and Electronic Engineering, Tianjin University of Technology, Tianjin 300384, China; zfmail163@163.com

**Keywords:** surface acoustic wave (SAW), molecularly imprinted, three-dimensional architecture graphene

## Abstract

In this paper, surface acoustic wave (SAW) sensors containing porous graphene/PVDF (polyvinylidene fluoride) molecularly imprinted sensitive membrane for DMMP gas detection were investigated. A 433 MHz ST-cut quartz SAW resonator was used to convert gas concentration changes into frequency shifts by the sensors. The porous graphene/PVDF film was fabricated on the sensor’s surface by using the tape-casting method. DMMP molecules were adsorbed on the porous structure sensing film prepared by the 2-step method to achieve the specific recognition effect. The sensitivity of the sensor could reach −1.407 kHz·ppm^−1^. The response time and recovery time of the SAW sensor with porous graphene/PVDF sensing membrane were about 4.5 s and 5.8 s at the concentration of 10 ppm, respectively. The sensor has good anti-interference ability to most gases in the air.

## 1. Introduction

Chemical warfare agents (CWAs) [1] are fast-acting lethal compounds even at low concentration levels. It is necessary to develop CWAs detection technology with high sensitivity, good selectivity, strong anti-interference ability, fast response and compatibility with the current Internet of things technology. Nerve gases [2] are the most dangerous agents of chemical warfare and mass destruction, can cause irreversible damage to the nervous system within seconds and are fatal if exposure occurs for even a few minutes. Therefore, it is very dangerous for researchers to directly study such gases’ sensing characteristics in the laboratory. Almost all studies examine simulants, which have no toxicity or less toxicity, but similar functional groups, structures and properties as nerve gases. DMMP [3,4] is a well-known nerve agent simulator, specifically of Sarin.

As a new and efficient recognition technology, molecularly imprinted technology [5,6] integrates materials science, polymer science, chemical engineering, biochemistry and other disciplines and can recognize specific analytes. Due to the characteristics of predetermination, recognition and practicability, it has been widely used in many fields, such as chromatographic separation [7,8], solid-phase extraction [9,10], biomimetic sensing [11], enzyme catalysis [12], clinical drug analysis [13] and so on. In addition, sensing films prepared by molecularly imprinted technology can also be integrated with electrochemical sensors and microelectronic sensors for detecting trace analytes.

Recently, surface acoustic wave (SAW) sensors [14,15,16] have been extensively used in liquid [17,18] and gas [19,20,21] detection because of their low cost, high precision, miniaturization, and compatibility with semiconductor technology. The SAW sensor consists of a sensing film and a conversion element (SAW resonator or delay line). A sensing film is the core of the SAW sensor. After it adsorbs the analyte to be measured, the SAW converter converts the adsorptions into frequency or phase shifts of the sensor to obtain the device’s response. Due to the high sensitivity and selectivity, the polymer [22,23,24], semiconductor [25,26] and graphene [27,28] sensing films have mostly been used in SAW sensors.

In this paper, we investigated a surface acoustic wave (SAW) sensor with porous PVDF layer sensing film based on molecularly imprinted recognition technology for simulant DMMP detection. A 433 MHz SAW one-port resonator based on ST-quartz substrate and aluminum interdigital transducers (Al-IDTs) was selected to convert the change of gas concentration into frequency shift.

## 2. Materials and Methods

### 2.1. Preparation of the SAW Sensor with a Porous Graphene/PVDF Sensing Layer

**Figure 1:**
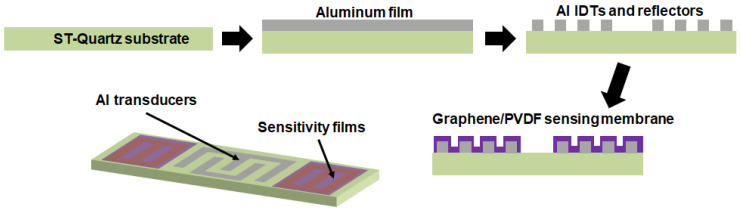
Schematic diagram of the fabrication process for the surface acoustic wave (SAW) sensor.

Figure 1 is a schematic diagram of the fabrication process for the one-port SAW resonator as a based device. The substrate was ST-quartz with a SAW velocity of 3158 m·s^−1^. The aluminum IDTs were fabricated using a standard UV-photolithography process. Each resonator consisted of an IDT with 160 pairs of electrodes and two reflectors on both sides of IDTs with 270 electrodes. The pitch and acoustic apertures were 1.8 μm and 172 μm, respectively. Moreover, the resonant frequency of the SAW devices was approximately 434.9 MHz. The sensing films were coated on both reflectors’ IDTs on the SAW resonator to reduce the propagation loss.

The porous graphene/PVDF sensing films were fabricated as the sensing membrane. First, 0.2 mmol of citric acid monohydrate (CAM), 0.02 mL of dimethyl methyl phosphonate (DMMP), 8 mg of graphene and 0.6 g of polyvinylidene fluoride (PVDF) powder was dispersed in 10 mL of N, N-dimethylformamide (DMF) with the assistance of ultrasonic dispersion to form CAM/DMMP/PVDF/graphene/DMF polymeric precursor solution. Then, 1 mL polymeric precursor solution was coated on the SAW resonator to obtain the porous film using a tape-casting method [29], followed by a drying treatment at 85 °C for 30 min in an oven.

**Figure 2:**
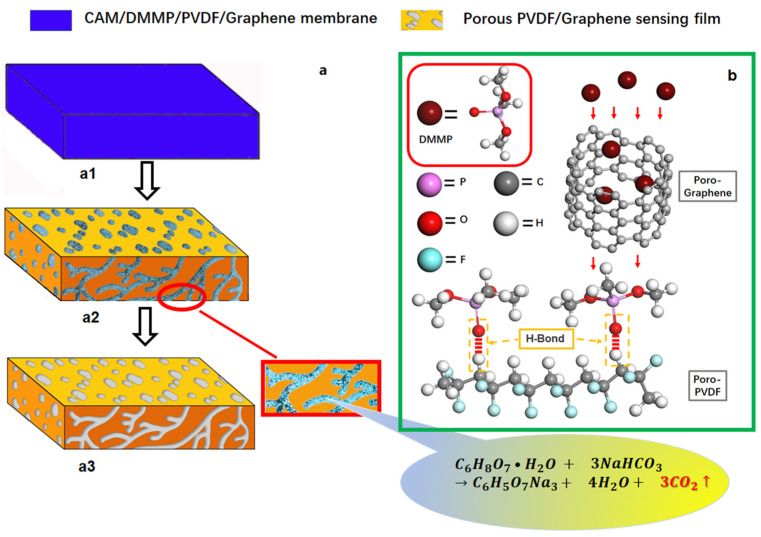
(**a**) Preparation process diagram of the sensing film, (**b**) gas adsorption and hydrogen bond formation diagram.

The preparation process of the sensing film on the SAW resonator is shown in Figure 2a. The graphene and recrystallized CAM micro/nanoparticles were uniformly dispersed in the cured graphene/PVDF/DMMP membrane (Figure 2(a1)). Next, the SAW device was immersed in the concentrated NaHCO_3_ (0.01 mol/L) aqueous solution for 1.5 h at 25 °C. Due to polymer swelling, the CAM nanoparticles near the surface of the sensing film will react with NaHCO_3_ at the interface between PVDF and graphene. Then, micro holes were formed with the emission of CO_2_. At the same time, DMMP on the inner wall of the micro-hole and the surface of the film was discharged with CO_2_ (Figure 2(a2)). Thus, the porous graphene/PVDF sensing films with DMMP molecular imprinting was formed. Finally, the devices were rinsed with ethanol and water to give the SAW sensors (Figure 2(a3)).

### 2.2. Characterization and Measurement

The morphologies of devices were characterized using scanning electron microscopy (FE-SEM; Merlin Compact, Zeiss, Oberkochen, Germany) and atomic force microscopy (AFM; Agilent 5500, Agilent Technologies, Santa Clara, CA, USA). The crystalline phases of materials were analyzed by X-ray diffraction (XRD; D/max-2500/PC, Rigaku, Tokyo, Japan). The responses of the SAW resonators and sensors were measured by a network analyzer (ENA-E5071C, Keysight, Santa Rosa, CA, USA).

**Figure 3:**
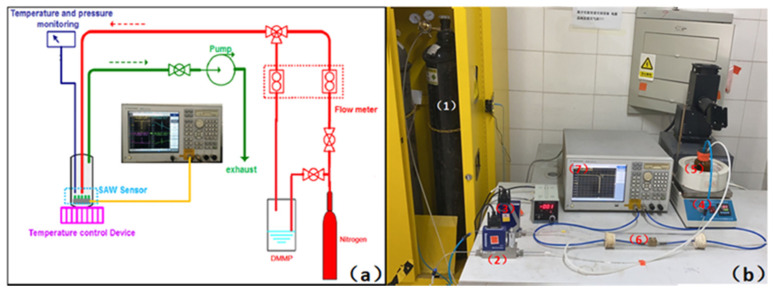
(**a**) Preparation process diagram of the sensing film, (**b**) DMMP measurement system.

For sensing vapors, a DMMP gas delivery and SAW measurement system was developed as Figure 3, which was included (1) standard N2 source, (2) massflow controller (MFC) for the mixture of N2/ DMMP, (3) MFC for N2, (4) heating device, (5) DMMP source, (6) prepared SAW sensor in a chamber, and (7) network analyzer. The nitrogen from the source was divided into two paths, one carrying the DMMP and the other only carrier to the sensor surface. Both were precisely controlled by MFC. The gas flow rate of the N2/DMMP path was maintained at 250 mL·min^−1^, and the flow rate of the pure nitrogen path can be adjusted. The flask containing DMMP is heated to 30 °C or 50 °C. Using the molar volume at different temperatures and flow differences between two gas channels, the concentration of the DMMP scan be achieved [30,31]. The SAW sensor was placed in a sealed chamber with a volume of about 500 mL. A pump was connected to the chamber’s outlet to realize the weak negative pressure in the test system, which could ensure the dynamic circulation of gas and the tightness of the system. Before testing, the SAW sensor, fixed on the test fixture, was placed in an N_2_ environment and preheated to 80 °C for 1 h to remove any gas absorbed on the sensing film.

## 3. Results and Discussion

### 3.1. Characterization and Measurement Results

**Figure 4:**
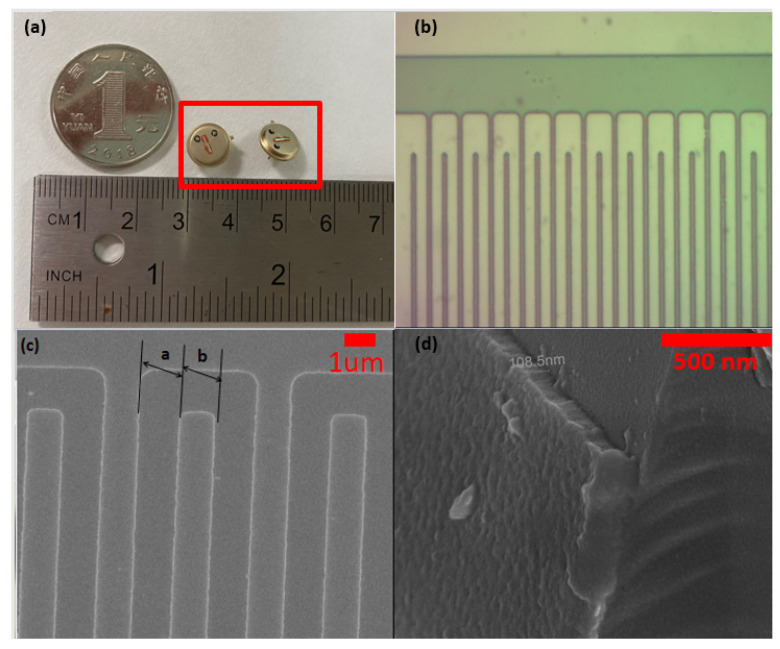
(**a**) One-port SAW resonators, (**b**) Optical microphotograph image of electrodes on bare SAW, (**c**) top view and (**d**) cross-sectional SEM images of interdigital transducers.

Figure 4a is the image of a one-port SAW resonator fabricated by a standard integrated circuit manufacturing method. Figure 4b,c shows the OM and SEM images of IDTs, which confirm that the IDTs finger width and gap are equal to 1.6 um, respectively. Figure 4d is the cross-sectional SEM images of IDTs, showing that the thickness of aluminum electrodes is about 108.5 nm.

**Figure 5:**
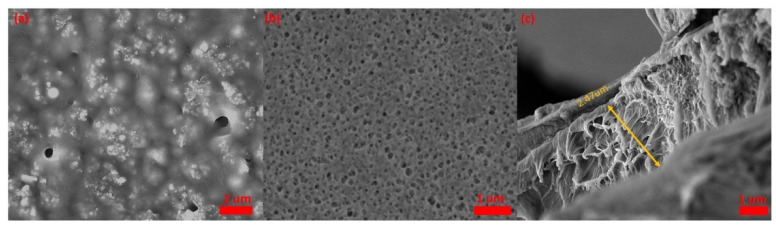
(**a**) SEM image of graphene/PVDF/CAM/DMMP film, (**b**) SEM image of porous graphene/PVDF film, (**c**) thickness of graphene/PVDF sensing film.

**Figure 6:**
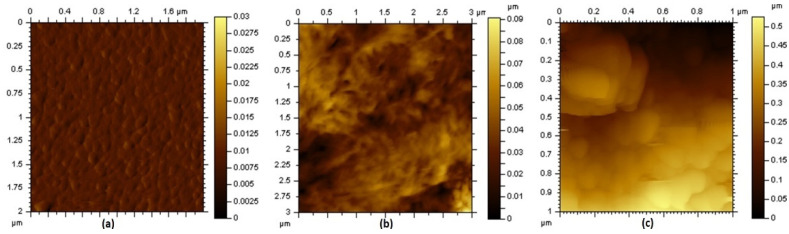
AFM image of (**a**) Al IDTs, (**b**) PVDF film and (**c**) porous graphene/PVDF sensing film.

**Figure 7:**
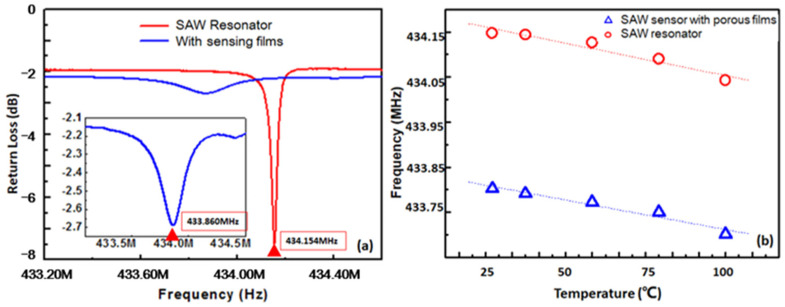
(**a**) Frequency characteristics of the SAW resonators (red) and SAW sensors with sensing films (blue), (**b**) TCF of the SAW resonators (red) and SAW sensors with sensing films (blue).

Figure 5a is the SEM images of graphene/PVDF/CAM/DMMP membrane, showing that the graphene and recrystallized CAM micro/nanoparticles were uniformly dispersed in the film. After immersion in the NaHCO_3_ solution, the recrystallized CAM precipitated in the form of carbon dioxide and formed a porous structure on the surface of the sensing film (Figure 5b). The thickness of the sensing film was about 2.47 μm (Figure 5c). Figure 6 shows AFM images of the Al IDTs, PVDF/DMMP film and porous graphene/PVDF sensing film. The roughness (RMS) of the IDT electrodes on one-port SAW resonator, PVDF/DMMP /SAW, and porous graphene/PVDF /SAW was 1.14 nm, 10.9 nm and 119 nm, respectively. With the formation of the porous structure sensing film, the roughness of the sensor surface increased significantly, which led to the S_11_ of the device decreasing from −8.21 dB to −2.68 dB (Figure 7a). Moreover, after the sensing film was fabricated on the reflective grating, the resonant frequency of the sensor showed a negative frequency shift of about 294 kHz due to the mass-loading effect [32].

The temperature coefficient of frequency (TCF), which is an important parameter to characterize the temperature stability of the SAW sensor, was observed in the range of 25–100 °C. As shown in Figure 7b, the preparation of the sensing film did not lead to significant changes in the temperature stability of the device. The TCF of the bare SAW resonator and SAW sensor with porous graphene/PVDF sensing membrane was −1.4305 ppm/°C and −1.5624 ppm/°C.

### 3.2. Static Responses of DMMP Sensors

**Figure 8:**
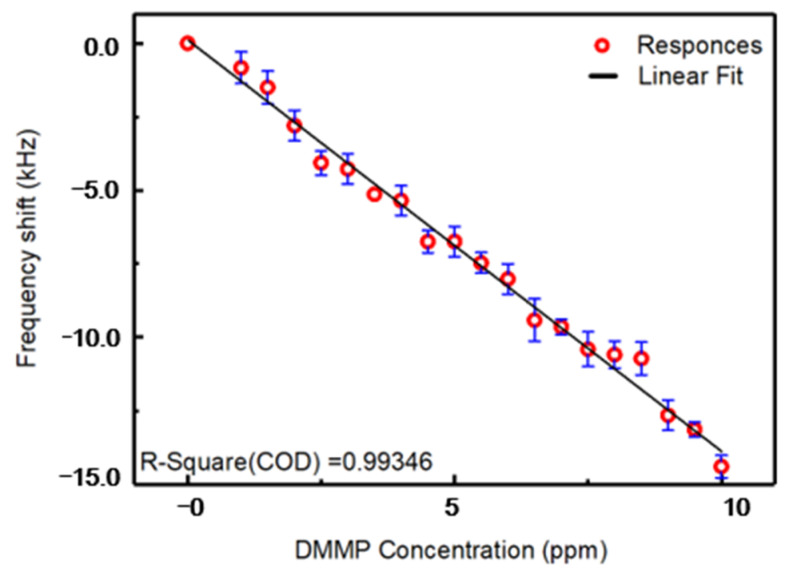
Responses of the sensor to different concentrations of DMMP in nitrogen.

Figure 8 shows the static responses of the SAW gas sensor with different DMMP concentrations in the range from 0 to 10 ppm. The sensor was tested more than 10 times at each concentration value. The raw data, statistical analysis and residual plots are shown in Table S1 and Figure S2. The response of the SAW sensor decreased rapidly with increasing DMMP concentration in the range from 0 to 10 ppm. The sensitivity of the sensor, which can be obtained from the slope of the linear fitting (solid black line in Figure 8), was about −1.407 kHz/ppm (R = 0.99346). The linear-regression equation, parameters, statistics and R-value are shown in Figure S3.

### 3.3. Dynamic Responses and Sensing Mechanism

The frequency shift of the SAW sensors based on the electric loading effect can be correlated with the sheet conductivity and the surface capacitance per length of the sensing film using the following relationship [33,34]:

$$\frac{\Delta f}{f_{0}}\;=\;-\left(\frac{K^{2}}{2}\right)\Delta \left[\frac{\sigma_{S}^{2}}{\sigma_{S}^{2}+v_{0}^{2}C_{S}^{2}}\right]$$

where *v*_0_ is the effective SAW velocity of the device, *K*^2^ is the electromechanical coupling coefficient of the piezoelectric substrate, *σ_S_* is the sheet conductivity, and *C_S_* is the surface capacitance per length. In the process of adsorbing DMMP gas, *C_S_*, which is determined by the structure of the device, remains constant. The porous graphene and PVDF film could both absorb DMMP gas and resulted in a decrease of resonant frequency due to the shift of the sheet conductivity. The sheet conductivity of porous graphene/PVDF film was about 6.1 × 10^−3^ S/cm under pure N2 and 2.7 × 10^−3^ S/cm under 10 ppm DMMP gas.

**Figure 9:**
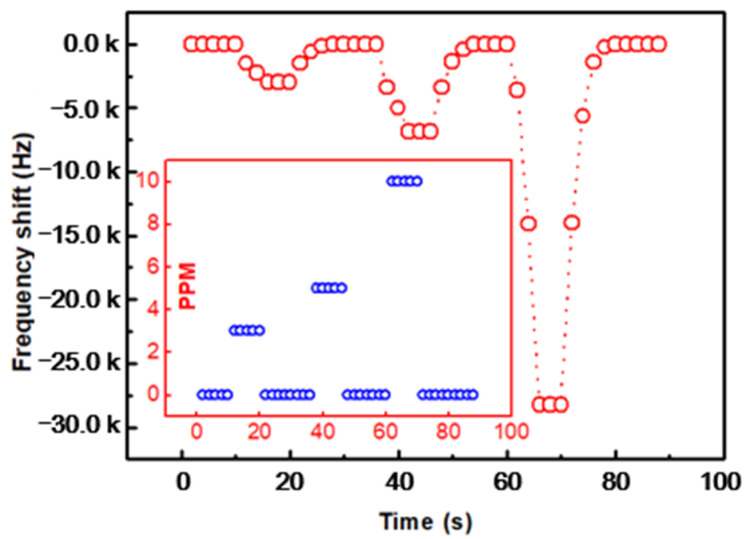
Dynamic response of the graphene/PVDF SAW sensor for DMMP.

As shown in Figure 9, the dynamic responses of the prepared SAW sensors with porous PVDF sensing film to fast DMMP concentration (0, 3, 5, 10 ppm) changes were investigated. The blue bubble was the DMMP concentration–time curve. The gas concentration was determined by the ratio of nitrogen and DMMP, while 0 ppm is pure nitrogen with equal gas flow. The response time and recovery time were defined as the time required for the frequency shift to achieve 10% and 90% of the change for the adsorption and desorption processed, respectively [35]. It can be seen from Figure 9 that the response time of the sensor was clearly faster than the recovery time. For the concentration of 3 ppm, 5 ppm and 10 ppm, the response time was about 2.9 s, 3.6 s, and 4.5 s, and the recovery time was about 3.7 s, 4.9 s, 5.8 s, respectively. When DMMP gas was introduced into the sensitive membrane, the molecularly imprinted sensitive membrane could quickly capture the analyte. Moreover, the hydrogen bond structure between DMMP and PVDF needs a certain gas flux to desorb when the gas concentration decreased. Figure 2b shows the gas adsorption and hydrogen bond formation diagram. In the multilayer sensing film, PVDF was used to protect the metal interdigital electrode, and porous graphene could effectively increase the specific surface area of the sensing film and increased the adsorption of the gas to be measured. When the sensitive membrane adsorbs DMMP gas, DMMP molecules pass through the porous structure, the O atom in the P = O group of DMMP and the H atom of PVDF form an H-bond [36] structure, which achieves specific recognition. The synergetic effect between PVDF and porous graphene was proven by the enhancement of sensitivity.

### 3.4. Selectivity and Stability

**Figure 10:**
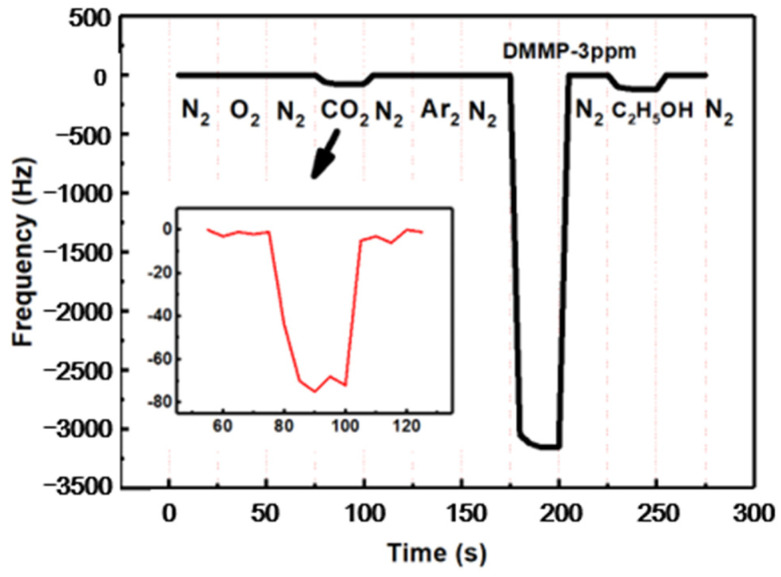
Selectivity of the SAW DMMP sensor.

**Figure 11:**
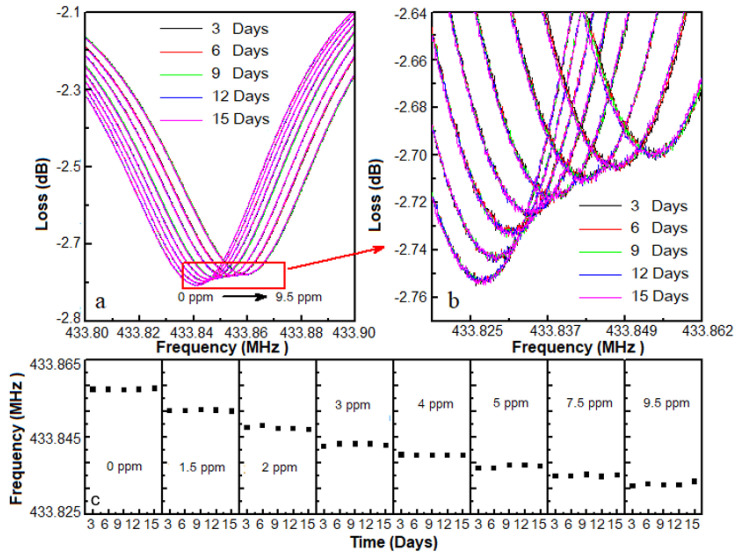
(**a**) Stability of the proposed SAW sensor; (**b**) frequency response curves of the sensor for various DMMP concentrations; (**c**) stability of the sensor stored at ambient conditions.

Figure 10 shows the selectivity of the SAW DMMP sensor. The SAW sensor did not have a marked response to pure N_2_, O_2_ and Ar. Although the responses could be interfaced by pure CO_2_ and ethanol gases, it was also far less than DMMP of 3 ppm. These results suggest that due to the preparation of molecularly imprinted sensitive membrane, the sensor had strong selectivity for DMMP. Furthermore, as shown in Figure 11, the response frequencies for different DMMP concentrations were recorded for 15 days to verify the stability of the sensors. When the time of storage increased, the frequency shifts of the porous graphene/PVDF SAW sensor were nearly constant.

## 4. Conclusions

In this paper, a DMMP gas sensor based on the SAW device with a porous graphene/PVDF molecularly imprinted sensing membrane was developed. The sensing membrane with a porous structure could improve the adsorption capacity of DMMP gas. Due to the molecularly imprinted recognition technology used in the sensing film, the SAW sensor, which had an excellent anti-interference ability, could detect DMMP specifically. The sensitivity of the sensor could reach −1.407 kHz·ppm^−1^. The response time of the SAW sensor with porous graphene/PVDF molecularly imprinted sensing membrane was faster than 4.5 s, and the recovery time was faster than 5.8 s at the concentration of 10 ppm. Furthermore, the developed SAW sensor could also be used for real-time and online monitoring of nerve gas.

## Supplementary Materials

The following are available online at https://www.mdpi.com/article/10.3390/mi12050552/s1, Figure S1: Responses of the bare SAW and sensor with sensitivity film for TCF, Figure S2: Residual plots for the data of Figure 8, Table S1: Statistical Analysis of the Experimental Data.
