# Supplementary material for: Surface Acoustic Wave DMMP Gas Sensor with a Porous Graphene/PVDF Molecularly Imprinted Sensing Membrane
Source: Micromachines (Basel). 2021 May 12;12(5):552. doi: 10.3390/mi12050552 (PMC8152042; doi:10.3390/mi12050552)
Supplement: Supplementary file 1 [file micromachines-12-00552-s001.zip › micromachines-1203936-supplementary.pdf]

Supplementary Data

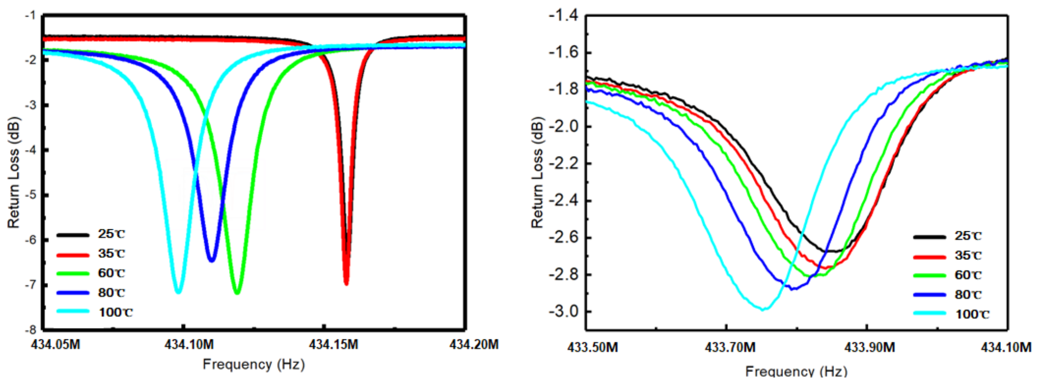

Figure S1. Responses of the bare SAW and sensor with sensitivity film for TCF

Table 1S. Statistical Analysis of the Experimental Data

PPM	N Total	Mean	Standard Deviation	Sum	Minimum	Median	Maximum
**0**	15	4.33629E8	0	6.50789E9	4.33859E8	4.33859E8	4.33859E8
**1**	15	4.33628E8	534.52248	6.50787E9	4.33857E8	4.33858E8	4.33859E8
**1.5**	14	4.33627E8	554.7002	6.07401E9	4.33857E8	4.33858E8	4.33859E8
**2**	15	4.33626E8	516.39778	6.50786E9	4.33857E8	4.33857E8	4.33858E8
**2.5**	15	4.33625E8	414.03934	6.50785E9	4.33856E8	4.33857E8	4.33857E8
**3**	15	4.33625E8	507.09255	6.50783E9	4.33855E8	4.33856E8	4.33856E8
**3.5**	15	4.33624E8	0	6.50783E9	4.33855E8	4.33855E8	4.33855E8
**4**	15	4.33623E8	507.09255	6.5078E9	4.33853E8	4.33853E8	4.33854E8
**4.5**	15	4.33622E8	377.96447	6.50778E9	4.33851E8	4.33852E8	4.33853E8
**5**	15	4.33622E8	516.39778	6.50776E9	4.3385E8	4.3385E8	4.33851E8
**5.5**	15	4.33621E8	351.86578	6.50775E9	4.3385E8	4.3385E8	4.33851E8
**6**	15	4.33621E8	516.39778	6.50774E9	4.33849E8	4.33849E8	4.3385E8
**6.5**	15	4.33619E8	723.74686	6.50772E9	4.33847E8	4.33848E8	4.33849E8
**7**	15	4.33619E8	258.19889	6.50769E9	4.33846E8	4.33846E8	4.33847E8
**7.5**	15	4.33618E8	593.61684	6.50766E9	4.33843E8	4.33844E8	4.33845E8
**8**	15	4.33618E8	457.73771	6.50761E9	4.3384E8	4.33841E8	4.33841E8
**8.5**	15	4.33618E8	560.61191	6.50759E9	4.33838E8	4.33839E8	4.3384E8
**9**	15	4.33616E8	516.39778	6.50755E9	4.33836E8	4.33837E8	4.33838E8
**9.5**	15	4.33616E8	258.19889	6.5075E9	4.33833E8	4.33833E8	4.33834E8
**10**	15	4.33614E8	377.96447	6.50745E9	4.33829E8	4.3383E8	4.33831E8

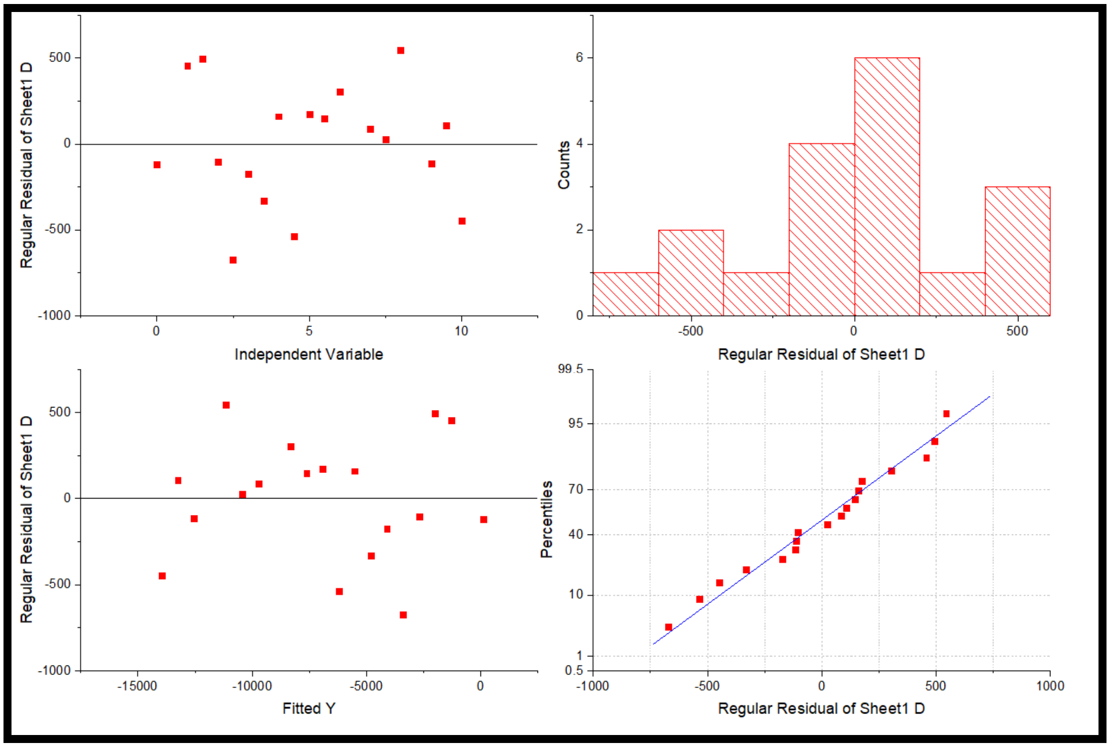

Figure S2. Residual plots for the data of Figure 8
